# Les sepsis intra-abdominaux diffus post-operatoires: aspects épidémiologiques, diagnostiques et thérapeutiques au Service de Chirurgie Générale du CHU Aristide Le Dantec de Dakar

**DOI:** 10.11604/pamj.2014.17.204.3111

**Published:** 2014-03-15

**Authors:** Alpha Oumar Touré, Mamadou Cissé, Ibrahima Ka, Madieng Dieng, Ibrahima Konaté, Ousmane Ka, Cheikh Tidiane Touré

**Affiliations:** 1 Service de Chirurgie Générale, CHU Aristide Le Dantec, Dakar, Sénégal

**Keywords:** Sepsis, postopératoires, péritonites, Sénégal, Sepsis, Postoperative, peritonitis, Senegal

## Abstract

Les sepsis intra-abdominaux diffus postopératoires (SIADPO) ont encore une fréquence alarmante. Ils mettent rapidement en cause l'intégrité des grandes fonctions. Le but de cette étude était d’évaluer leur prise en charge. Il s'agissait d'une étude rétrospective descriptive sur 10 ans (janvier 2000 à décembre 2009) portant sur 45 cas de SIADPO. Nous avons étudié les aspects épidémiologiques, diagnostiques, thérapeutiques et pronostiques. Il s'agissait de 45 cas dont 25 hommes et 20 femmes avec un sex-ratio de 1,25. L’âge moyen des patients était de 34 ans avec des extrêmes de 20 et 70 ans. Le SIADPO survenait au décours d'une intervention septique en urgence dans 68,8% des cas. Le délai moyen de diagnostic était de 10 jours. Les signes cliniques étaient dominés par les troubles du transit (80%), la douleur abdominale (77,7%), la fièvre (66,7%), le météorisme abdominal (33%). Une hyperleucocytose a été retrouvée dans 60% des cas. Le liquide intra-abdominal était polymicrobien. Tous les patients ont bénéficié d'une laparotomie xipho-pubienne dans les 72 heures. Nous avons noté 82,2% de péritonites secondaires notamment post-opératoires et 17,8% de péritonites persistantes. Les étiologies étaient dominées par le lâchage de suture digestive ou gynécologique (66,7% des cas). La stomie digestive a été le geste le plus fréquemment réalisé (41%). La guérison est survenue chez 75,5%. La morbidité opératoire était de 42% faite de suppuration pariétale (10 cas), de fistule entérocutanée (6 cas), d’éviscération (2 cas). La mortalité était de 24,5% en rapport avec le retard diagnostique et les défaillances multi-viscérales. Les interventions septiques en urgence sont les plus grandes pourvoyeuses de SIADPO. La mortalité reste encore élevée en rapport avec la défaillance viscérale. La précocité du diagnostic et de la réintervention conditionnent ainsi le pronostic.

## Introduction

Malgré une maîtrise des techniques chirurgicales, et les progrès réalisés dans les domaines de la réanimation, les sepsis intra-abdominaux diffus postopératoires (SIADPO) ont encore une fréquence alarmante. Elles restent un problème grave grevé d'une lourde mortalité [1, 2]. Ils mettent rapidement en cause l'intégrité des grandes fonctions et imposent, à coté de l'acte chirurgical judicieusement indiqué et réalisé, la mise en oeuvre intensive de mesures de réanimation. Ces sepsis comportent, en gros, deux entités représentées par les péritonites persistantes et les péritonites post-opératoires. La quasi-totalité des études concernant ce sujet ont été menées dans les pays industrialisés. Nos conditions d'exercice avec, le plateau technique chirurgical déficient, les services de réanimation sous-équipés, le niveau socio-économique bas des patients, sont autant de paramètres qui ont motivé une étude rétrospective portant sur 45 cas de sepsis intra-abdominaux diffus post-opératoires colligés au service de Chirurgie Générale de l'hôpital Aristide Le Dantec sur une période de 10 années. Le but de notre travail est de faire ressortir les particularités épidémiologiques, diagnostiques et thérapeutiques dans nos conditions.

## Méthodes

Il s'agit d'une étude rétrospective descriptive allant du 1er janvier 2000 au 31 décembre 2009, soit une période de 10 ans. Cette étude s'est déroulée au sein du Service des Urgences Chirurgicales de l'Hôpital Le Dantec. Nous avons inclus, dans notre étude, tous les patients qui ont été pris en charge pour péritonites post-opératoires par lâchage de sutures viscérales ou persistantes durant cette période. Soixante-quatorze dossiers médicaux étaient concernés parmi lesquels 29 étaient inexploitables. Il s'agissait de 25 hommes (55, 6%) et de 20 femmes (44,4%). Le sex-ratio était de 1,25. L’âge moyen était de 34 ans avec des extrêmes de 20 et 70 ans. Nous avons étudié les paramètres épidémiologiques tels que le terrain, les antécédents et les données de l'intervention initiale. Les aspects diagnostiques ont été déterminés à partir du délai diagnostique, de la symptomatologie clinique, des données de la biologie et des examens radiologiques, et de l'existence de défaillances viscérales. Nous avons également évalué le traitement.

## Résultats

Vingt-et-un patients étaient porteurs d'une pathologie chronique comme le montre le Tableau 1. Trente-huit patients (84,4%) des patients ont été opérés en urgence et 7 (15,6%) en chirurgie réglée. Le degré de septicité de ces interventions a été objectivé en fonction de la classification d'Altemeier. Les interventions septiques (Classes III et IV) représentaient 68,8% des cas avec 44,4% pour la classe III et 24,4% pour la classe IV.

**Tableau 1:** Terrain des patients

Terrain	Nombre de cas	Pourcentage
Hypertension artérielle	7	33,3%
Cardiopathie ischémique	1	4,7%
Diabète de type 2	2	9,6%
Néoplasie digestive	11	52,4%
Total	21	100%

Des incidents opératoires ont été décrits à type de: brèches iléales accidentelles après adhésiolyse dans 3 cas soit 6,7% (1 péritonite appendiculaire et 2 péritonites par perforation iléale); plaie oesophagienne dans 1 cas (2,2%) lors d'une fundoplicature pour hernie hiatale. Le délai moyen entre la 1ère intervention et le diagnostic de SIADPO était de 10 jours avec des extrêmes d'1 et de 28 jours. Le diagnostic a été posé entre le 1er et le 5ème jour dans 37,8% des cas (n=17) et entre le 6ème et le 10ème jour post-opératoire dans 24,4% des cas (n=11).

La symptomatologie clinique, peu spécifique et très variable était dominée par la douleur (77,7%) et la fièvre (66,7%). Par ailleurs, la dénutrition était retrouvée chez 51,1% des patients (Tableau 2).

**Tableau 2:** Symptomatologie des sepsis intra-abdominaux diffus post-operatoires

Signes cliniques	Nombre de cas	Pourcentage (n=45)
Dénutrition	23	51,1
Douleur	35	77,7
Vomissements	20	44,4
Arrêt du transit	11	24,4
Diarrhée	5	11
Fièvre	30	66,7
Hypothermie	1	2,2
Tachycardie	19	42,2
Polypnée	22	48,9
Chute de la diurèse	9	20
Hypersécrétion gastrique	4	8,9
Etat de choc	10	22,2
Défense	6	13,3
Météorisme	15	33,3
Eviscération	4	8,9
Ecoulement anormal	12	26,7
Nécrose de stomie	5	11
Troubles psychiques	1	2,2

L'examen de la numération et de la formule sanguines avait permis de retrouver: une hyperleucocytose supérieure à 12 000 éléments/mm 3 dans 60% des cas (n=27) avec une prédominance neutrophile; une anémie à un taux d'hémoglobine inférieur à 9g/dl dans 42,2% (n=19); une leucopénie avec 3900 éléments/mm3 dans 2,2% des cas (n=1); une hyperplaquettose supérieure à 500000/mm3 dans 6,6% des cas (n=3); une thrombopénie dans 4,4% des cas (n=2).

Une hypoglycémie mesurée à 0,45g/l a été notée chez 1 patient (2,2%). L'ionogramme sanguin était perturbé dans 4 cas (8,9%). Une insuffisance rénale fonctionnelle avec un taux de créatinine sanguine supérieure à 15 mg/l était notée dans 2 cas (4,4%). Une hypoprotidémie inférieure à 50g/l a été objectivée chez 16 patients (35,5%). Des troubles de la crase sanguine ont été retrouvés dans 4,4% des cas (n=2) avec un taux de prothrombine < 60%.

L'examen bactériologique des prélèvements intra-abdominaux étaient multibactériens chez 15 patients (33,3%). Le spectre microbien était polymophe et les associations de germes aérobies ont prédominé avec 73,3% des résultats. Les germes retrouvés sont évocateurs de la flore digestive avec une présence de bacilles gram négatif et anaérobies (Tableau 3). Un taux de résistance de 70,7% a été objectivé parmi ces germes tandis que 29,3% d'entre elles étaient multi-résistantes.

**Tableau 3:** Répartition des germes isolés

Germes	Nombre	Pourcentage (n=15)
**Aérobies**		
*Escherichia coli (Bacille Gram négatif)*	8	53,3
*Streptococcus (cocci gram positif)*	3	20
*Staphylococcus (cocci gram positif)*	5	33,3
*Klebsiella (bacille gram négatif)*	5	33,3
*Proteus (bacille gram négatif)*	1	6,7
*Pseudomonas (bacille gram négatif)*	3	20
*Enterobacter (bacille gram négatif)*	1	6,7
*Morganella (bacille gram négatif)*	1	6,7
**Anaérobies**		
*Bacteroides*	4	26,7
Association aérobies	11	73,3
Association aérobies- anaérobies	4	26,7

La radiographie de l'abdomen sans préparation (ASP) a été réalisée chez 20 patients soit 44,4% des cas. Elle a permis d'objectiver une grisaille diffuse dans 10 cas, des niveaux hydro-aériques dans 7 cas, un croissant interhépato-diaphragmatique dans 3 cas. L’échographie abdominale réalisée chez 3 patients soit 6,6% des cas a montré respectivement une distension intestinale, une collection pelvienne et une collection hétérogène du cul-de-sac de Douglas et des loges sous-phréniques. La radiographie pulmonaire avait montré, chez 1 patient, une opacité pulmonaire basale droite. Le scanner n'a été réalisé chez aucun de nos patients. Des défaillances viscérales ont été mises en évidence chez 20 patients (44,4%). Les défaillances hémodynamiques (42,2%), cardiaque (8,9%) et respiratoire (6,7%) prédominaient. Quinze patients ont présenté une association de 2 défaillances au moins.

Nos patients ont tous été pris en charge dans les 72 heures suivant le diagnostic. Durant les 24 heures après le diagnostic de SIADPO, 64,4% des patients ont été réopérés. Tous nos patients ont bénéficié d'une préparation pré-opératoire par un remplissage vasculaire à base de sérum physiologique dont la quantité variait en fonction du poids et de la diurèse; l'objectif étant d'obtenir une diurèse d'au moins 75ml/heure. Des flacons de macromolécules ont été perfusés aux patients présentant un état de choc, soit dans 22,2% des cas. Une transfusion de sang total a été effectuée chez 19 patients (42,2%). Du plasma frais congelé a été administré chez 2 patients (4,4%).

Nos patients ont tous bénéficié d'une laparotomie médiane sus et sous-ombilicale. Le liquide péritonéal était purulent dans 64,4% des cas (n=29), louche dans 22,2% des cas (n=10) et séro-hématique dans 13,4% des cas (n=6),. En classant les étiologies, nous avons mis en évidence 82,2% de péritonites post-opératoires (n=37) et 17,8% de péritonites persistantes (n=8). Parmi les péritonites post-opératoires, le lâchage de suture ou d'anastomose a représenté 66,7% (n=30) des étiologies (Tableau 4). Dans 2 cas de péritonite persistante, nous avons retrouvé une lésion méconnue lors de l'intervention initiale à savoir une perforation d'ulcère duodénal méconnue et une appendicite méconnue.

**Tableau 4:** Répartition des etiologies de sepsis intra-abdominaux diffus post-operatoires

Etiologies	Nombre	Pourcentage
Péritonites post-opératoires et secondaires		
Lâchage de suture ou d'anastomose	30	66,7
Nécrose ou rétraction de stomie	5	11,1
Nécrose utérine	1	2,2
Rupture de kyste pancréatique	1	2,2
Péritonites persistantes		
Ulcère perforé méconnu	1	2,2
Appendicite rétro-coecale méconnue	1	2,2
Absence de lésion viscérale	6	13,4
Total	45	100

La stomie digestive a été le geste le plus fréquent soit 41% des traitements étiologiques (n=16). La toilette péritonéale, avec un volume moyen de sérum de 3,75 litres, suivie d'un drainage de la cavité abdominale était systématique pour tous les patients en plus du traitement de la cause du sepsis. Le drainage a été fait par une lame de Delbet dans 80% des cas (n=36) et par drain tubulé dans 20% des cas (n=9). La fermeture pariétale a été faite plan par plan dans 80% des cas (n=36), par des points totaux dans 20% des cas (n= 9).

Lors des interventions d'urgence digestive soit 66,7% des cas (n= 30), l'association Ampicilline - Gentamycine - Métronidazole a été instituée par voie parentérale en 1ère intention puis modifiée au profit du céfotaxime (1g×2/jour). En réalité, le céfotaxime a été institué comme traitement probabiliste dans 100% des cas des SIADPO dés le diagnostic posé. L'antibiothérapie a été adaptée aux résultats de l'antibiogramme dans 33,3% des cas (n=15).

La durée moyenne de séjour des patients a été de 33, 7 jours avec des extrêmes de 9 et 78 jours. Vingt-quatre patients, soit 53,3% des cas, ont été transférés au service de réanimation après l'intervention. Leur durée de séjour moyen était de 3 jours avec des extrêmes d'1 et 7 jours. Le rétablissement de la continuité digestive a été réalisé ultérieurement chez tous les patients porteurs de stomie. La guérison est survenue chez 34 des patients soit 75,5% des cas.

Une morbidité de 37,8% (n=17) a été observée avec 10 cas de suppuration pariétale (22,2%) ayant bien évolué sous une antibiothérapie adaptée et soins locaux; 4 cas de fistule entéro-cutanée (8,8%) ayant évolué vers une fermeture spontanée; 2 cas d’éviscération (4,4%) aux suites simples après réfection pariétale; et 1 cas d'escarre fessière superficielle (2,2%) ayant bien évolué grâce à des pansements à la biaffine.

Onze décès ont été notés soit 24,5% des cas. Parmi ces patients, 6 étaient âgés de plus de 50 ans. Parmi les patients ayant séjourné en réanimation, 25% (n=6) sont décédés. Le nombre de décès survenus rapporté au délai de diagnostic de SIADPO montre 100% de décès pour les cas diagnostiqués entre le 16ème et le 20ème jour; 50% de décès pour les cas diagnostiqués entre le 21ème et le 25ème jour; et 100% de décès pour les cas diagnostiqués entre le 25ème et le 30ème jour. Les patients présentant une association de défaillances ont prédominé. Deux défaillances ont été retrouvées chez 54,5% des patients décédés (n=6) et 3 défaillances chez 27,3% des patients décédés (n=3). La mortalité était de 100% chez les patients avec 3 défaillances viscérales et de 43% chez les patients présentant 2 défaillances.

## Discussion

Dans cette étude, l’âge moyen des patients était de 34 ans avec un taux de 42% pour la tranche de 20 à 30 ans. Cissé et coll. rapportent, dans leur étude, 60% de cas de péritonites post-opératoires avec un âge compris entre 17 et 36 ans et Mignonsin, une prédominance des SIADPO chez les patients ayant un âge entre 11 et 30 ans [3, 4]. Les mêmes auteurs ont observé une proportion masculine de 75-80% des cas [3, 4]. Les taux retrouvés par notre étude ne semblent pas superposables même si une légère prédominance masculine avec un taux de 55, 6% a été notée. L'inclusion des sepsis secondaires à une chirurgie obstétricale à ce travail peut expliquer cette légère discordance.

Les interventions qui prédisposent le plus à un sepsis post-opératoire sont celles effectuées dans un contexte initial septique. Il a été ainsi rapporté un accroissement des infections post-opératoires de 0,1% après chirurgie propre à 6,5% en cas d'une chirurgie septique [1, 5, 6]. Les interventions «contaminées» (classe 3 d'Altemeier), par exemple les traumatismes pénétrants, ont un taux d'infection de 20%. Enfin, les interventions «sales» (classe 4 d'Altemeier), sont associées à une forte contamination, avec des taux d'infection de 35% [7]. Dans notre étude, 44,4% des actes initiaux ont été des actes de classe 3 d'Altemeier et 24,4% étaient des actes de classe 4.

Les conditions d'intervention (situation d'urgence) et le terrain du patient (corticothérapie, dénutrition, maladie inflammatoire du tube digestif …) sont également évoqués dans la littérature comme facteurs favorisants [1, 2]. 84,4% des interventions initiales ont été effectuées en urgence dans ce travail.

Cependant, les conditions locales (zone irradiée ou cancéreuse) et la difficulté du geste chirurgical paraissent les éléments les plus importants dans la survenue d'un sepsis post-opératoire [1, 2, 5]. Onze patients de cette étude présentaient un cancer digestif.

Une étude évaluant la fréquence des infections post-opératoires lors d'appendicectomies a rapporté une incidence de complications infectieuses plus élevée chez les opérateurs juniors que chez les chirurgiens séniors [8]. Le niveau de l'opérateur n'a pas été étudié comme paramètre dans cette étude. Mais la majorité des interventions en urgence, dans notre contexte, est effectué par des chirurgiens en formation. Les péritonites persistantes retrouvées pourraient relever d'une faute technique du fait de leur manque d'expérience.

Les SIADPO sont tardivement reconnues, classiquement entre le 5ème et le 7ème jour post-opératoire. Un second pic correspond aux complications retardées constatées au-delà de la seconde semaine [1]. Dans notre étude, le diagnostic de SIADPO a été posé dans les 10 premiers jours post-opératoires dans 62,2% des cas. Souvent, les signes extra- abdominaux donnent un tableau trompeur qui pourrait expliquer le retard diagnostique observé au-delà du 15ème jour chez 15,6% des patients de cette étude responsable d'un retard dans la prise en charge notamment en ce qui concerne la décision de reprise chirurgicale. Dans cette étude, la douleur abdominale et la fièvre ont été les signes dominants. Ces chiffres sont superposables à ceux rapportés par d'autres auteurs [3, 9]. La fièvre et la douleur sont des signes dominants constamment retrouvés dans les SIADPO [10]. L'installation ou la persistance d'une fièvre ou plus rarement d'une hypothermie dans la période post-opératoire d'une chirurgie abdominale doit faire rechercher un foyer infectieux intra-abdominal [11]. Il faut, au préalable, avoir éliminé les autres causes telles que le paludisme dans nos zones d'endémie [7]. Les signes abdominaux sont difficiles à interpréter en raison de l'iléus post-opératoire et de la douleur abdominale habituelle chez l'opéré récent. Une diarrhée précoce est un bon signe de désunion anastomotique alors que l'hyper-sécrétion gastrique est une conséquence directe de l'infection péritonéale [1, 7, 12].

Les signes extra-abdominaux sont précoces, polymorphes et trompeurs. Ils sont constitués d’éléments pronostiques présageant d'une éventuelle défaillance viscérale [1, 3, 4, 9]. Parmi nos patients, 22,2% avaient présenté un état de choc et 20% avaient une oligurie. Ces signes ont le tort d'orienter vers des causes médicales retardant la prise en charge et grevant le pronostic vital.

Les examens biologiques usuels sont généralement décevants. Une hyperleucocytose a été retrouvée dans 60% des cas. Ce signe est banal dans la période post-opératoire mais il doit attirer l'attention lorsqu'il persiste au-delà du 3ème jour post-opératoire ou qu'il est de forte concentration (> 15-20000/mm3) [1]. Les autres signes biologiques comme les troubles électrolytiques, les perturbations de la fonction rénale, les troubles de la crase sanguine et autres, ne permettent pas d'orienter le diagnostic avant le stade de défaillance viscérale.

Les prélèvements intra-abdominaux qui ont été effectués dans notre étude ont été polymicrobiens comme l'ont rapporté plusieurs auteurs [1, 3, 10, 11, 13]. Le caractère multi-microbien de ces bactériémies est très évocateur de SIADPO et les souches sont souvent multirésistantes [1, 10, 14]. Les germes aérobies représentent 70 à 90% des souches isolées [2]. La majorité des prélèvements bactériologiques effectués dans notre contexte sont mal conservés ou ne sont pas déposés au laboratoire. Ceci pourrait expliquer le faible nombre de résultats bactériologiques retrouvés dans notre étude.

Même si aucun de nos patients n'a bénéficié d'un scanner, il est l'examen le plus performant avec une sensibilité de 95% et une spécificité de 90% même si elle présente l'inconvénient de devoir déplacer le patient [15]. Le scanner injecté permet une différenciation entre les viscères et l’épanchement intra-péritonéal [15]. Nous avons surtout utilisé l'ASP qui se révèle difficile d'interprétation en post-opératoire [1].

Les étiologies des SIADPO sont dominées par le lâchage de sutures digestives et génitales et celui d'anastomoses digestives dans 66,7% des cas dans cette série comme rapporté par Lévy [13]. Dans cette série, le contexte d'urgence de l'intervention initiale dans lequel ces sutures ont été effectuées explique la fréquence de cette étiologie, en plus de la contamination relative de la cavité abdominale comme le suggère Montravers [1, 13]. D'autres éléments tels que les incidents opératoires peuvent être incriminés. Les péritonites persistantes sont en rapport avec un défaut de toilette péritonéale. En effet, nous sommes souvent confrontés à des difficultés d'ordre économique car la fourniture de la quasi-totalité du matériel incombe aux patients. Il arrive qu'en certaines occasions la quantité de solutés disponible soit insuffisante pour assurer une toilette péritonéale de qualité.

Le traitement des SIADPO est essentiellement chirurgical mais il est associé à un volet médical incontournable. Celui-ci comporte 4 axes: la rééquilibration hydro-électrolytique, la lutte contre l'hypoxie, l'infection et la dénutrition [10]. Les principes de la prise en charge chirurgicale des SIADPO sont l’éradication de toutes les sources d'infection intra-abdominale impliquant une voie d'abord permettant l'exploration la plus complète possible afin d’être certain que l'on ne laisse aucune source d'infection persistante; le traitement de la source de l'infection par éradication du foyer infectieux ou en limitant le plus possible la récidive de l'infection; la réduction de l'inoculum bactérien avec toilette péritonéale et drainage, pour aider le péritoine à lutter contre l'infection. [7]. Cependant, la réintervention n'est pas indiquée chez tous les patients présentant un SIADPO. Il existe des critères de temporisation ou de réintervention chirurgicale [4]. La source de l'infection dans les péritonites post-opératoires doit être suturée, exclue ou réséquée. Une stomie a été réalisée dans 41% des cas de notre étude. Le lavage péritonéal au sérum physiologique est usuellement pratiqué en cas de péritonite avec au moins 6 litres de sérum chauffé. Mais aucune étude n'a prouvé l'efficacité de ce lavage péritonéal [16]. Au moindre doute quant à l’éradication du sepsis, il est préconisé de drainer la cavité abdominale [17]. Le drainage peut servir d’élément de surveillance par la quantité et l'aspect du liquide qui s'en extériorise [18]. Il peut avoir un effet bénéfique en favorisant l’évacuation de liquide résiduel. Il a permis, dans quelques cas, de diagnostiquer une fistule digestive. En cas de suites opératoires simples, il est généralement enlevé au bout de 5 jours.

La guérison est survenue chez 75,5% de nos patients. Ce taux est relativement élevé en raison de la précocité de la reprise chirurgicale (64,4% dans les 24 heures) et de l’âge jeune de nos patients. Chichom a obtenu des résultats similaires [19]. La durée moyenne d'hospitalisation de nos patients (33,7 jours) est en rapport avec la réalisation de stomies digestives et le rétablissement différé de la continuité. Une morbidité de 42,2%, se rapprochant de ceux antérieurement obtenus par Cissé et coll., a été observée dans notre série avec 10 cas de suppuration pariétale, 6 cas de fistule entéro-cutanée, 2 cas d’éviscération et 1 cas d'escarre fessière [3]. Onze de nos patients sont décédés soient 24,4% des cas. Il s'agit le plus souvent de plusieurs facteurs qui agissent simultanément. En effet, il existerait aussi une relation entre le délai opératoire et la mortalité. Nous avons constaté une mortalité élevée chez les patients dont le diagnostic de SIADPO a été retardé avec près de 100% de mortalité au-delà du 15ème jour suivant l'intervention initiale. L'existence de défaillances viscérales est le facteur prédictif de mortalité le plus constant dans les différentes séries [3, 20]. Dans cette présente étude, 9 des 11 patients décédés présentaient au moins 2 défaillances viscérales.

## Conclusion

Il convient, dès lors, de pêcher par excès, en préférant reprendre le patient atteint de SIADPO le plus tôt possible afin d'optimiser les chances de survie avant l'installation des défaillances multiviscérales. Les patients qui présentent des facteurs de risque de survenue de SIADPO doivent bénéficier d'une attention particulière. Des précautions seront prises aussi bien sur le plan chirurgical que celui de la réanimation pour guetter la survenue de complications en post-opératoire. Il faut surtout retenir que la prise en charge est médico-chirurgicale. Dans nos conditions d'exercice, chez un opéré récent qui ne va pas bien, il est préférable de le reprendre précocement après une réanimation optimale.
